# General practice recommendations for the topical treatment of psoriasis: a modified-Delphi approach

**DOI:** 10.3399/bjgpopen20X101108

**Published:** 2020-11-11

**Authors:** Diamant Thaçi, Pablo de la Cueva, Andrew E Pink, Ahmad Jalili, Siegfried Segaert, Kasper F Hjuler, Piergiacomo Calzavara-Pinton

**Affiliations:** 1 Institute and Comprehensive Center for Inflammation Medicine, University of Lübeck, Lübeck, Germany; 2 Department of Dermatology, University Hospital Infanta Leonor de Madrid, Madrid, Spain; 3 St John’s Institute of Dermatology, Guy’s and St Thomas’ NHS Foundation Trust, London, UK; 4 Department of Dermatology, Bürgenstock Medical Center, Obbürgen, Switzerland; 5 Consultant Dermatologist, Bonheiden, Belgium; 6 Department of Dermatology, Aarhus University Hospital, Aarhus, Denmark; 7 Department of Dermatology, University of Brescia, Brescia, Italy

**Keywords:** primary health care, recommendations, psoriasis, topical treatment, adherence, general practitioners

## Abstract

**Background:**

Although GPs are usually the first port of call for patients with psoriasis, there is a lack of consistent and up-to-date clinical recommendations for interventions for patients with mild-to-moderate disease.

**Aim:**

To provide practical recommendations for GPs to optimise psoriasis treatment with topical therapies in four key areas: patient identification; treatment decision making with topical theory; topical treatment outcomes; and optimising patient adherence.

**Design & setting:**

A consensus-seeking programme (modified-Delphi approach) was undertaken to assess the literature and develop recommendations for GPs, based on evidence and expert opinion.

**Method:**

Three dermatologists compiled 47 questions that were subsequently ranked and refined according to clinical relevance or importance using an online survey. Thereafter, 19 dermatologists from different European countries developed statements and clinical recommendations for the top seven ranked topical treatment and GP-relevant questions based on literature research and clinical experience. The final recommendations were based on 100% agreement among a final panel of seven experts.

**Results:**

The clinical effectiveness, fast onset of action, tolerability, cosmetic acceptability, and practicability of topical therapy, in addition to good physician—patient communication, are important for optimising patient adherence and maximising efficacy. Topical treatments combining corticosteroids and vitamin D analogues (administered as fixed combination) are well-established first-line treatments in mild-to-moderate psoriasis.

**Conclusion:**

Simple but detailed practical guidance is provided, which is formed from evidence and expert clinical recommendations, to assist GPs with the optimal use of topical agents based on efficacy, tolerability, disease severity, site of psoriasis, patient lifestyle and preferences, and intended duration of treatment.

## How this fits in

Topical treatments are indicated as first-line treatment options for patients with mild-to-moderate psoriasis. In order to maximise patient outcomes to topical treatments, tailored up-to-date guidance for GPs is needed. This guidance, based on evidence and expert clinical recommendations, provides practical recommendations for GPs, with the aim of optimising use of topical treatments and improving patient outcomes. Topics covered include: patient identification; treatment decision making with topical therapy; topical treatment outcomes; and optimising patient adherence.

## Introduction

Psoriasis is a chronic, immune-mediated, inflammatory skin disease, which affects around 2% of the Western population.^1,2^ The most common form, psoriasis vulgaris/plaque psoriasis, accounts for the majority of cases and is defined by recurrent scaly, itchy, erythematous plaques that usually affect the elbows, knees, scalp, sacral region, and intertriginous areas.^3,4^ Patients with psoriasis experience varying degrees of severity and disability, and the socioeconomic impact of psoriasis can cause decade-long impairments in quality of life (QoL).^3,5^ In many countries, most patients with mild-to-moderate psoriasis are managed in primary care by GPs.^6^

Approximately 80% of patients with psoriasis have localised disease, which can be treated with topical therapies.^7^ Currently approved topical treatments include corticosteroids, vitamin D analogues, combination corticosteroid and vitamin D, vitamin A derivatives (tazarotene), anthralin, and newer formulations of tar.^7^ Adherence rates to topical treatments are relatively low, varying from 50%–70%,^2,8^ with rates even lower (40%) for topical corticosteroids and in patients with severe psoriasis.^8^ Almost 50% of topical treatment prescriptions for psoriasis remain unfilled.^9^

While there are several guidelines available on the management of psoriasis, the majority of these focus on systemic treatments, and there is a lack of up-to-date practical recommendations specifically tailored for GPs regarding topical treatments and how they can help to maximise patient outcomes.^10–13^ This guidance seeks to provide clarity around, and support implementation of, treatment algorithms, and aid clinicians in optimising an individual's care. Clear guidance is provided in four key areas to support practical implementation: patient identification; treatment decision making with topical therapy; topical treatment outcomes; and optimising patient adherence. Guidance regarding the use of topical agents while transitioning to systemic treatments is also discussed.

## Method

This was a consensus-seeking programme using a modified-Delphi approach as outlined below. An expert group of seven dermatologists (DT, PC, AEP, AJ, SS, KFH, and PGC-P; the ‘core group’) from across Europe participated in a programme to devise recommendations for the use of topical treatments. With the exception of KFH, the experts had originally been selected to participate in a LEO Pharma-funded international steering committee in which the need for this initiative was highlighted. KFH was contracted by LEO Pharma to support with the initiative. All of the experts were identified based on their extensive psoriasis clinical expertise. They were selected from different European countries in order to provide a comprehensive evaluation of possible local differences in topical therapeutic approaches to psoriasis. Supplementary Figure S1 provides an overview of the programme. The ‘working group’ (PB, PGC-P, and PC), nominated by the core group, identified 47 potentially relevant questions this initiative could seek to address. A survey to prioritise these questions and determine which were the most clinically important and relevant to address was shared with a wider group of expert dermatologists (selected by the core group based on dermatology expertise), and a final list of seven questions relating to topical treatment and relevant to GPs was generated (Table 1). The wider expert group included the authors of this manuscript and 12 additional practising dermatologists from 10 countries, thus providing a breadth of clinical experience from many healthcare systems.

**Table 1.** Final questions

Which patients are candidates for topical psoriasis treatment? What factors inform the selection of one topical treatment over another for mild-to-moderate psoriasis? What outcomes should a physician or patient expect from topical treatment and within what timeframe? How long should topical treatments be used for? What are the drivers of treatment switching? What features of a topical treatment maximise adherence? What simple instructions can be shared with patients regarding the use of topical treatments to maximise efficacy and adherence?

The working group, supported by medical writers from Leading Edge Medical Communications, conducted literature searches (details of the literature searches are provided in Table 2 and search strings used for each question are provided in Supplementary Table S1) to address each question and the working group alone developed draft statements summarising the literature. Available evidence obtained for each literature statement was assigned a level of evidence, graded in line with the Oxford Centre for Evidence Based Medicine.^14^ Additionally, the working group addressed evidence gaps by providing practical clinical recommendations based on their clinical experience and expert opinion. A second survey of the wider expert group obtained feedback on the draft statements and recommendations. Responders were asked if they agreed with the draft summary statements and clinical recommendations, to rank the latter according to clinical importance, and to suggest any additional clinical recommendations. The final summary statements and clinical recommendations were then generated based on 100% agreement among the core group and are presented herein.

**Table 2.** Summary of literature searches

Database searched	PubMed (MEDLINE)
Date of search	April 2018
Limits	Languages: EnglishSpecies: humansPublication date: last 5 years
Manual searches	Abstracts:European Academy of Dermatology and Venereology meetings 2016 and 2017American Academy of Dermatology meetings 2016, 2017, and 2018
Inclusion criteria	Systematic reviews, meta-analyses, clinical practice guidelines, and reports of clinical trials, randomised clinical trials, cohort studies, registry analyses, and case series
Exclusion criteria	Non-EnglishNon-humanReferences older than 5 yearsReferences that are not focused on topical treatments

## Results

Clinical recommendations are provided below for each of the questions. Summary statements regarding the rationale behind each of these, as well as additional tips, are provided in Supplementary Table S2.

### Patient identification and profile

#### Question 1: Which patients are candidates for topical psoriasis treatment?

- Topical treatment (selected according to specific body sites and the clinical phenotype of disease) is generally a first-line treatment approach in all patients treated by GPs.
- Patients with moderate or severe disease receiving systemic therapies or phototherapy may benefit from concomitant topical therapies because residual disease can cause considerable impact on health-related quality of life (HRQoL).

### Drivers of treatment decision

#### Question 2: What factors inform the selection of one topical treatment over another for mild-to-moderate psoriasis?

**Table 3.** Advantages, limitations, and practical considerations of the most commonly used topical formulations for psoriasis

**Vehicle formulation**	**Advantages**	**Limitations**	**Typical use**
Ointment	Occlusive effect, potentially improves penetration and efficacy.^26^ Hydrating effect.Simple formula, often preservative-free.	Greasiness as no evaporation or absorption.^27^Can be time-consuming and less cosmetically appealing.^28^	Dry, thick, scaly plaques for body.Not for hair-bearing skin.
Cream	Less greasy, more spreadable than ointment.^27^Some hydrating effect.Cosmetically acceptable.	Less occlusive effect, decreased penetration and efficacy compared with ointment.	All body areas.Hair-bearing areas including scalp with correct application technique.
Gel; water- or lipid-based	Easy application, easy to spread.^27^Cosmetically acceptable.	Minimal or no occlusion.Minimal hydration.	All body areas, especially well-suited for hair-bearing skin.
Aerosol foam; hydroalcoholic or emollient-based	Easy application, easy to spread.Usually no preservatives.Some skin hydration if emollient-based.^29^Cosmetically acceptable.	Minimal occlusion and hydrating effects (hydroalcoholic).Some greasiness if emollient-based foam.Risk of stinging, irritation, and dryness if alcohol-based.^29^	All body areas, especially hair-bearing areas (if not emollient-based).
Lotion	Easy application, not greasy.Cosmetically acceptable.Cooling skin effect.^27^	No occlusive effect.No or minimal hydration.	Hair-bearing areas.Not optimal for the treatment of thick, scaly plaques in other areas.
Solution; alcohol- or water-based	Easy to spread.Not greasy.No residue.Cosmetically acceptable.	Stinging, irritation, and dryness if alcohol-based.Needs to be shaken (inhomogeneous solution).	Hair-bearing areas.Avoid alcoholic solutions if they crack or excoriate skin.
Shampoo	Developed for scalp use.Not greasy.Cosmetically acceptable.	No occlusive or hydrating effect.Complex application procedure.Short contact time with skin.	Hair-bearing areas, especially scalp with dense hair growth.

Individual patient preferences, drug characteristics, and active pharmaceutical ingredient (API)-specific features have not been taken into account.

**Table 4.** Appropriate topical treatments for psoriasis by site and expected time to treatment outcome

**Site of psoriasis**	**Treatment^a^**	**Expected time frame to outcome**
Body	Vitamin D analogues (dose should not exceed 5 mg per week)^30,31^ Combination vitamin D analogue and corticosteroid Potent or super-potent corticosteroids (short-term use only) Salicylic acid plus corticosteroid	2–4 weeks
Hands and feet	Potent or super-potent corticosteroids (short-term use only) Combination vitamin D analogue and corticosteroid Salicylic acid plus corticosteroid Occlusion overnight at start of treatment (plastic glove or sock) Propylene glycol plus corticosteroid^b^	4 weeks
Scalp	Potent or super-potent corticosteroid Combination vitamin D analogue and corticosteroid Salicylic acid plus corticosteroid Tar plus salicylic acid plus sulphur	4 weeks
Face	Low- or mid-potency corticosteroid Topical calcineurin inhibitor	2 weeks
Perianal or genital area and skin folds (axillae, inframammary, or inguinal region)	Low- or mid-potency corticosteroid Topical calcineurin inhibitor^c^	2 weeks

This information is based on the expert opinion and clinical experience of the authors and provides a suggested option for a given clinical scenario to contribute to an informed decision; it does not advise that a given treatment is warranted. ^a^Treatments are not listed in any particular order. ^b^Certain countries only (for example, UK and Switzerland). ^c^2–3 days of corticosteroids before switching to topical calcineurin inhibitors (owing to burning).

- Choice of topical agent should be based on the site (Table 3 and Table 4), extent and features of psoriasis, such as level of hyperkeratosis, redness, thickness, presence of inflammation, severity of itch, and pain.
- Practical factors should be considered, for example, formulation (Table 3), and discussed with your patient to ensure suitability.
- If prescribing corticosteroids, the right potency for the area affected should be chosen (see Table 4 for guidance).
- There are systemic safety issues with use of potent corticosteroids on large skin areas that are more pronounced if the area is occluded.
- Initial use of low-potency (for example, hydrocortisone cream 1%) or underdosed topical corticosteroids should be avoided as treatment is unlikely to be successful and this could adversely affect adherence.
- Special consideration needs to be given to certain patient subpopulations, that is, children, pregnant women, and breastfeeding mothers. Prescribing information should be checked before using topical treatments for them (see Supplementary Table S2 for further guidance).

**Figure 1.**
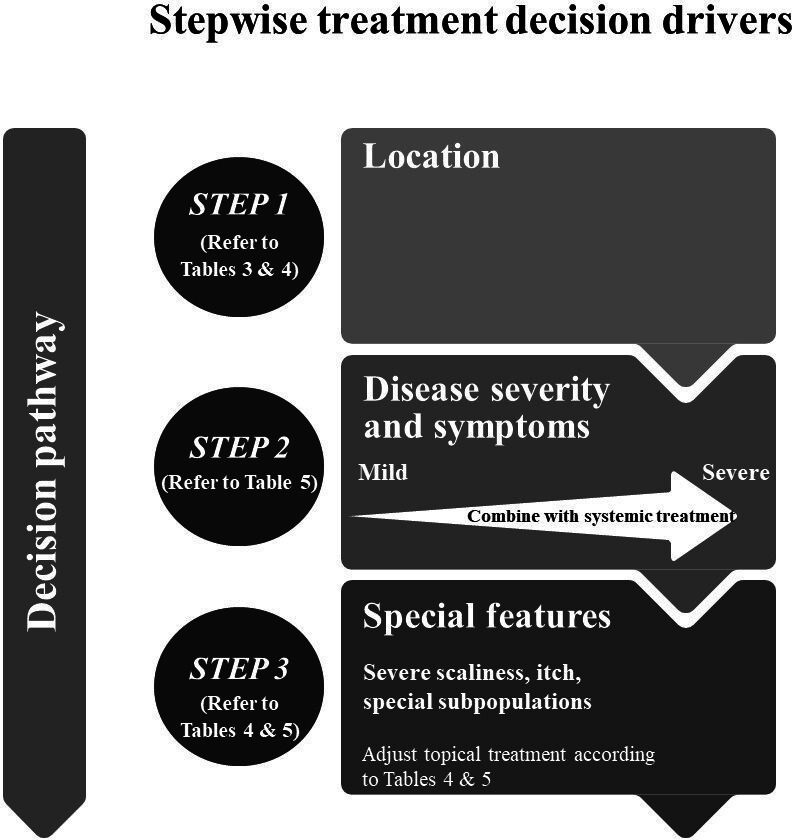
Topical treatment algorithm

Figure 1 is provided as guidance to aid treatment decision making. It highlights stepwise-treatment decision drivers based on disease location, severity and symptoms, and other considerations, such as presence of itch or patient subpopulations. Disease severity is generally assessed using: Physician Global Assessment (PGA); Body Surface Area (BSA); or Psoriasis Area and Severity Index (PASI), which rate the quality (redness, thickness, and scaliness) and/or surface area of the plaques. For practical tips on estimating BSA see Supplementary Table S2, section 1. Potent and super-potent topical corticosteroids are effective, but their long-term use is limited by concerns about side effects.^1^ For patients receiving topical corticosteroids, particularly the highest potency, it is recommended that disease severity is monitored and an alternative topical treatment is switched to (for example, calcipotriene plus betamethasone dipropionate [Cal/BD] fixed-dose combination) after 2 weeks (if no response) and a maximum of 4 weeks. Calcipotriol and calcipotriol or Cal/BD are marketed by LEO Pharma, the sponsor of this work, under the tradenames (UK) Dovonex (calcipotriol ointment), Enstilar (Cal/BD foam), and Dovobet (Cal/BD gel and ointment). The fixed-dose Cal/BD aerosol foam is preferable because it leads to higher skin penetration of the active ingredients and has superior efficacy compared with ointment or gel formulation^15,16^ and monotherapies.^4^

Emollients play a basic role in the topical treatment of psoriasis,^17^ and there is some evidence of improvement of psoriasis with keratolytic compounds used as monotherapy or in combination with other therapies, either during or before the use of active drugs.^18^

It is believed there is currently no ‘one-size-fits-all’ treatment pathway for patients with mild-to-moderate psoriasis and no preferred sequence of treatments. While treatment decision making should be individualised to the patient, choice of treatment and formulation can be based on an individual’s key symptom(s). Table 5 has been designed to support treatment decision making based on symptoms of psoriasis and should be used in conjunction with Tables 3 and 4 to factor in the location of the disease.

**Table 5.** Clinical effectiveness of topical treatments on psoriasis symptoms

**Key feature of psoriasis**	**Corticosteroid**	**Vitamin D analogue**	**Cal/BD foam fixed-dose combination^a^**	**Topical calcineurin inhibitor**	**Salicylic acid**	**Urea**	**Tar^b^**
Redness	+++	+/++	+++	+/++^c^	0	0	+
Scaling	+	+/++^d^	++	0	+++	++	+
Thickness or infiltration	++	+/++	+++	+	0	0	++
Itching	++/+++	0	+++	++	0	+	++

Symbols represent clinical effectiveness, where + = somewhat effective and +++ = very effective; 0 represents no or minimal impact on the symptom. This information is based on the expert opinion and clinical experience of the authors and provides a suggested option for a given clinical scenario to contribute to an informed decision; it does not advise that a given treatment is warranted. Only the most commonly used vehicles are included in the Table. It is not intended as a comprehensive list. Clinical effectiveness is based on the active ingredients; overall effectiveness in the real world is also driven by patient adherence and the vehicle plays an important part in patient behaviour. ^a^Cal/BD is marketed by LEO Pharma, the sponsor of this work, under the tradenames (UK) Enstilar (Cal/BD foam) and Dovobet (Cal/BD gel and ointment). ^b^Use of tar is country-specific: coal-tar preparations are used in the UK^32^ and elsewhere as part of the Goeckermann regimen^33^; however, tar is rarely used in Switzerland, Austria, or Scandinavia. ^c^++ = when used on face. ^d^Cream = +; ointment = ++.

### Optimal outcomes of topical treatment

#### Question 3: What outcomes should a physician or patient expect from topical treatment and within what time frame?

- Satisfactory improvement (for example, changes in redness, scaling, thickness or plaques, and the size of the area involved) should be observed by the patient within 2 weeks. For some symptoms, such as itch, improvement may be felt as early as a few days.^19,20^ At around 4 weeks, a marked improvement (for example, clear or almost clear outcome) should be expected, even if further improvement can still be seen after 4 weeks. If there is no clinically relevant improvement after 4 weeks, adherence should be assessed (including consumption) and treatment switched if the patient is fully adherent (Table 4).
  - If residual redness is seen, without plaque elevation or desquamation, treatment can be tapered.
- Patient expectations should be carefully managed to ensure they are realistic about:
  - how well the medication may work;
  - how soon they should expect to see an improvement in symptoms;
  - side effects that may occur; and
  - any additional treatment that may be required beyond 4–8 weeks.

### Topical treatment duration

#### Question 4: How long should topical treatments be used for?

Table 4 details the suggested length of treatment based on the expected time frame to achieving outcome (from expert experience).

- A topical treatment should be used (except potent and super-potent corticosteroids) at the minimum effective frequency for as long as it provides continuous improvement or disease control, and is safe and well tolerated with minimal side effects.
- Regardless of plaque location, potent corticosteroids should not be used for longer than 6 weeks without interruption. If remission is not achieved in this time frame, the patient should be referred to a dermatologist, as the diagnosis may need to be reconsidered and/or systemic treatment options considered. In the meantime, less potent corticosteroids or alternative topical drugs can be used. If necessary, after an interruption of at least 6–8 weeks, treatment with potent corticosteroids can be recommenced. This statement is based on clinical experience of dermatologists, as there is no published consistent clinical and experimental evidence to support this statement; the potency of different corticosteroids is provided in Supplementary Table S3.
- Super-potent steroids, such as clobetasol, should not be used daily for >2 weeks.
- Combination therapies containing topical corticosteroids and salicylic acid (if not contraindicated) can be used for as long as the strength of the corticosteroid component allows, provided that scaling has been significantly reduced.
- If long-term topical treatment of psoriasis is required, vitamin D3 analogues and their combinations are recommended.
- Psoriasis should be treated to minimum disease activity regardless of the site of the disease.

The frequency of flares, if ascertainable, can be used to inform whether a patient requires long-term management with topicals. There are different approaches to treatment regimens; for example, option one: used daily for a number of weeks until clear or almost clear and then switched to ‘as needed’, during which time the patient can retreat when needed. Option 2: treated daily for a number of weeks, followed by gradual tapering; for example, every second day for 2 weeks, then twice a week for 2 weeks, once a week for 2 weeks, and then the treatment is paused. If, however, mild psoriasis activity increases at any stage, tapering should be ceased and an appropriate interval should be established at which the disease is at an acceptable level of activity.

In addition to the ‘as needed’ and ‘tapering’ approaches detailed above, maintenance treatment with topical therapies, using reduced potency and/or frequency, can be considered. For example, consider the option of applying topical therapies on two specified days of the week, as agreed in collaboration with the patient.

### Drivers of treatment switching, escalation, or sequencing

#### Question 5: What are the drivers of treatment switching?

- Consider switching topical treatments if:
  - poor or no improvement is seen within 4 weeks;
  - patient is dissatisfied and wishes to explore other options;
  - patient has continued psoriasis-related impairment of HRQoL;
  - the treatment is poorly tolerated;
  - patient’s lifestyle or circumstances change; and/or
  - newer interventions with better benefit or risk profiles become available.
- If poor adherence is suspected and ascertained, the patient should be worked with to try to improve it before switching, unless a treatment-specific factor is the reason for poor adherence.

### Drivers of adherence

#### Question 6: What features of a topical treatment maximise adherence?

a. The following features of a topical treatment maximise adherence (Table 1 and Table 2):

- Clinical effectiveness
  - The appearance of plaques should be examined to assess disease severity, which is defined as severe (thick and scaly with a lot of erythema), moderate (moderate in its scaling, thickness, and redness), mild or almost clear (possibly still pink and barely perceptible without real scaling), and clear (completely clear without any erythema), as well as improvement in other symptoms, such as itching.^7^
- Fast onset of action
  - Tell the patient that the effect can be expected within 1–2 weeks of treatment and that they must remain patient if it takes longer (that is, up to 6–8 weeks of treatment).
- Tolerable side effects
  - Treatments that minimise tolerability problems (for example, burning and itching) and do not cause obvious skin atrophy are preferred.
- Cosmetically acceptable
  - Formulation, areas of application, and patient preference should be considered (Table 2).
- Practicability
  - Some patients may prefer the simplicity and practicability of a fixed-dose combination regimen, which allows reduced frequency of application versus monotherapies.
  - Choose an easy treatment regimen that is also preferred by the patient, for example, once-daily application.

Corticosteroids (alone or in combination) are often the topical treatment of choice, but ‘corticosteroid-phobia’, which has been described as an irrational fear towards steroids, is a contributing factor in treatment non-adherence.^21^ Rather than prescribe other (often less effective) steroid-free treatments, patients should be reassured that the safety of modern topical corticosteroids is well established when used appropriately.^21^

### Topical treatment initiation

#### Question 7: What simple instructions can be shared with patients regarding the use of topical treatments to maximise efficacy and adherence?

- Ensure you have been as specific as possible. Written instruction should contain: name of product, dosing, frequency, body location, and length of treatment.
- Available written instructions should be used, for example, a compact pocket card, from patient associations or other trusted sources, which you have reviewed and are in line with your recommendations.
- Where appropriate, family members should be involved to support the patient with their treatment. This is particularly relevant if the disease is located on the scalp or back, or if the patient has reduced mobility or severe obesity.

For further guidance on optimising doctor–patient communication, see Supplementary Table S4.

### Considerations beyond topical treatments

Patients with moderate-to-severe psoriasis should be referred to specialists, as they may require systemic therapy or phototherapy. The experts agreed that patients should remain on topical therapies while transitioning to systemics or while waiting for an appointment with a specialist.

## Discussion

### Summary

This expert and evidence-driven consensus programme, which used a modified-Delphi approach, was intended to provide practical recommendations for GPs for the use of topical therapies in psoriasis, with a view to improving treatment decision making and patient outcomes. This could be particularly useful given the absence of updated guidelines on topical treatments for psoriasis.

### Strengths and limitations

The study endeavoured to seek contribution on clinical best practice from a range of countries, and while substantial differences in clinical practice are not anticipated, recommendations may not fully reflect current best practice in all clinical settings around the globe.

### Comparison with existing literature

The majority of patients with psoriasis suffer from mild-to-moderate disease and most of them do not require systemic therapy;^7^ instead, the mainstay of their treatment is topical therapy and, where clinically relevant, phototherapy.^22,23^ Advantages of topical treatments over biologic or traditional systemic treatments include: wide availability, lower costs, lack of serious safety issues (traditional systemic treatments are often discontinued owing to safety and tolerability concerns);^24^ as well as the fact that topical agents can empower patients to take control of the psoriatic disease.^22,23^

A key barrier to treatment success of a topical treatment is patient non-adherence.^7^ Healthcare professionals play a pivotal role in instructing and advising their patients to help improve adherence to treatment,^7^ asking questions to ensure the patient’s understanding of benefits and the use of topical therapies as prescribed. The relapsing-remitting disease course of psoriasis means that long-term management is often required, with regular follow-up to assess response and make any necessary treatment adjustments. GPs and practice nurses are in key positions to directly influence a patient’s perception of psoriasis, and to clearly explain the importance of continuing treatment.^7^ Substantial and ongoing research in topical treatment has led to: improved efficacy by enhancing drug delivery and bioavailability of active ingredients, without compromising safety; enhanced convenience of application; and increased variety of available formulations.^4^ New topical formulations with greater efficacy and more convenient application could lead to better adherence and provide subsequent long-term maintenance of a disease-free state.^4^ Reformulations of well‐known active ingredients, for example, calcipotriol or Cal/BD as foam, have been associated with improved clinical outcomes,^25^ improved cosmetic acceptance, better tolerability, and reduced frequency of application.

### Implications for practice

These recommendations, based on evidence and on expert opinion, were designed to aid GPs in optimising and individualising psoriasis treatment, with regards to choice and optimal use of topical agents.
